# Long noncoding RNA SBF2-AS1 contributes to the growth and metastatic phenotypes of NSCLC via regulating miR-338-3p/ADAM17 axis

**DOI:** 10.18632/aging.103332

**Published:** 2020-09-25

**Authors:** Qi Chen, Sheng Min Guo, Hou Qiang Huang, Guo Ping Huang, Yi Li, Zi Hui Li, Run Huang, Lu Xiao, Chun Rong Fan, Qing Yuan, Si Lin Zheng

**Affiliations:** 1 Nursing Department, The Affiliated Hospital of Southwest Medical University, Luzhou 646000, Sichuan, China; 2 Rehabilitation Department, The Affiliated Hospital of Southwest Medical University, Luzhou 646000, Sichuan, China; 3 Laboratory Medicine, Zigong Maternal and Child Care Service Centre, Zigong 643000, Sichuan, China; 4 School of Basic Medicine, Southwest Medical University, Luzhou 646000, Sichuan, China

**Keywords:** lncRNA, SBF2-AS1, NSCLC, miR-338-3p, ADAM17

## Abstract

Non-small cell lung cancer (NSCLC) is a type of refractory malignant lung cancer with a high rate of metastasis and mortality. Currently, long non-coding RNA (lncRNA) SBF2 Antisense RNA 1 (SBF2-AS1) is considered as a biomarker for a variety of tumors. However, the function of SBF2-AS1 in the growth and metastasis of NSCLC needs to be further studied. In this study, we revealed that SBF2-AS1 was overexpressed in NSCLC tissues compared with that in normal tissues. SBF2-AS1 silencing restrained the growth and aggressive phenotypes of NSCLC cell *in vitro*. Consistently, SBF2-AS1 knockdown hindered the growth of NSCLC cell in nude mice. The following luciferase reporter gene assay and RNA immunoprecipitation (RIP) assay suggested the relationship between miR-338-3p and SBF2-AS1. The rescue experiments showed that miR-338-3p inhibitor abolished SBF2-AS1 silencing caused inhibition on the growth, migration and invasiveness of NSCLC cell. The luciferase reporter assay and immunoblotting assay validated that A Disintegrin and Metalloprotease 17 (ADAM17) was a target of miR-338-3p. In addition, SBF2-AS1 positively regulated the level of ADAM17 through sponging for miR-338-3p. Finally, we revealed that SBF2-AS1 contributed to the proliferation and metastatic phenotypes of NSCLC cell via regulating miR-338-3p/ADAM17 axis.

## INTRODUCTION

Lung cancer is the mainly cause of cancer-related mortality worldwide. Although the great advance in the therapeutic methods, including targeted therapies and immunotherapy, the prognosis for patients with advanced non-small cell lung cancer (NSCLC) stages remain very poor. The primary reason for the poor outcomes in patients with NSCLC is the presence of systemic metastases. Hence, it is critical to investigate novel molecule biomarkers for combating the metastasis of NSCLC.

Long non-coding RNAs (lncRNAs) are a class of noncoding RNAs with longer than 200 nucleotides in length. Increasing reports have proved that lncRNAs are commonly dysregulated in multiple tumor types. Simultaneously, an increasing number of reports have confirmed that lncRNAs serve as oncogenes or suppressors through controlling the progression of malignant tumors. For instance, lncRNA Colorectal Neoplasia Differentially Expressed (CRNDE) stabilized by Heterogeneous Nuclear Ribonucleoprotein U Like 2 (hnRNPUL2) facilitates the growth and metastasis of colon cancer cell by activating Ras/MAPK signaling pathway [1]. LINC00511 promotes the malignant phenotypes of clear cell renal cell carcinoma via sponging miRNA-625 and thereby increasing cyclin D1 expression [2]. LncRNA UICLM promotes the metastasis of colon cancer cell through acting as a competing endogenous RNA (ceRNA) for miRNA-215 to regulate ZEB2 expression [3]. LncRNA SNHG7 facilitates the mobility and invasive capacity of liver cancer cell via modulating miR-122-5p and Ribosomal Protein L4 (RPL4) [4]. Recently, lncRNA SBF2-AS1 has been identified as an oncogene in various types of cancer [5–9].

Notably, promising evidences have implied that lncRNAs, including SBF2-AS1, serve as ceRNAs to modulate genes expressions through competitively binding with microRNAs (miRNAs) in cancers. Investigations of the precise roles and molecular mechanisms of lncRNA-miRNA-mRNA crosstalk are necessary to improve the clinical outcomes of patients with cancer. In gastric cancer, lncRNA SBF2-AS1 plays oncogenic roles via regulating the miR-302b-3p-E2F Transcription Factor 3 (E2F3) axis [10]. Silencing of SBF2-AS1 impairs the growth, migration ability and invasiveness of human osteosarcoma cell through upregulating miRNA-30a and inhibiting the expression of Forkhead Box A1 (FOXA1) [11]. Moreover, lncRNA SBF2-AS1 acts as a ceRNA sponging miRNA-142-3p to participate in gemcitabine resistance in human pancreatic cancer via upregulating Twinfilin Actin Binding Protein 1 (TWF1) [12]. However, the potential function of lncRNA SBF2-AS1 during the development of NSCLC needs to be more deeply explored.

MiRNAs, which are noncoding RNAs, interact with the 3’-UTR of target genes to regulate the expressions of cancer suppressors or oncogenes. Previous investigations verify that miRNAs are involved into numerous pathological processes, for example cancers. Currently, miR-338-3p has been reported to function as a cancer inhibitor in many tumor types. For example, miR-338-3p suppresses the metastasis of ovarian carcinoma cell through suppressing proliferation and epithelial-mesenchymal transition (EMT) enhanced by MET Transcriptional Regulator MACC1 (MACC1) [13]. Previous report also indicates that miRNA-338-3p restrains the proliferation and induces the apoptosis of NSCLC cell via modulating sphingosine kinase 2 (SPHK2) [14]. In NSCLC, lncRNA colorectal neoplasia differentially expressed (CRNDE) serves as an oncogene and play crucial roles in NSCLC development by sponging miR-338-3p [15]. But the potential regulatory mechanism behind the dysregulation of SBF2-AS1 and miR-338-3p in the growth and metastatic-traits phenotypes of NSCLC is still unclear.

Herein, we revealed the precise interaction between lncRNA SBF2-AS1 and miR-338-3p, which regulated the growth and aggressiveness of NSCLC cell by modulating its target gene ADAM Metallopeptidase Domain 17 (ADAM17). Based on these results, a novel regulatory signaling axis composed of SBF2-AS1/miR-338-3p/ADAM17 was illuminated in the progression of NSCLC.

## RESULTS

### SBF2-AS1 is correlates with the clinicopathological features in human NSCLC

**Figure 1:**
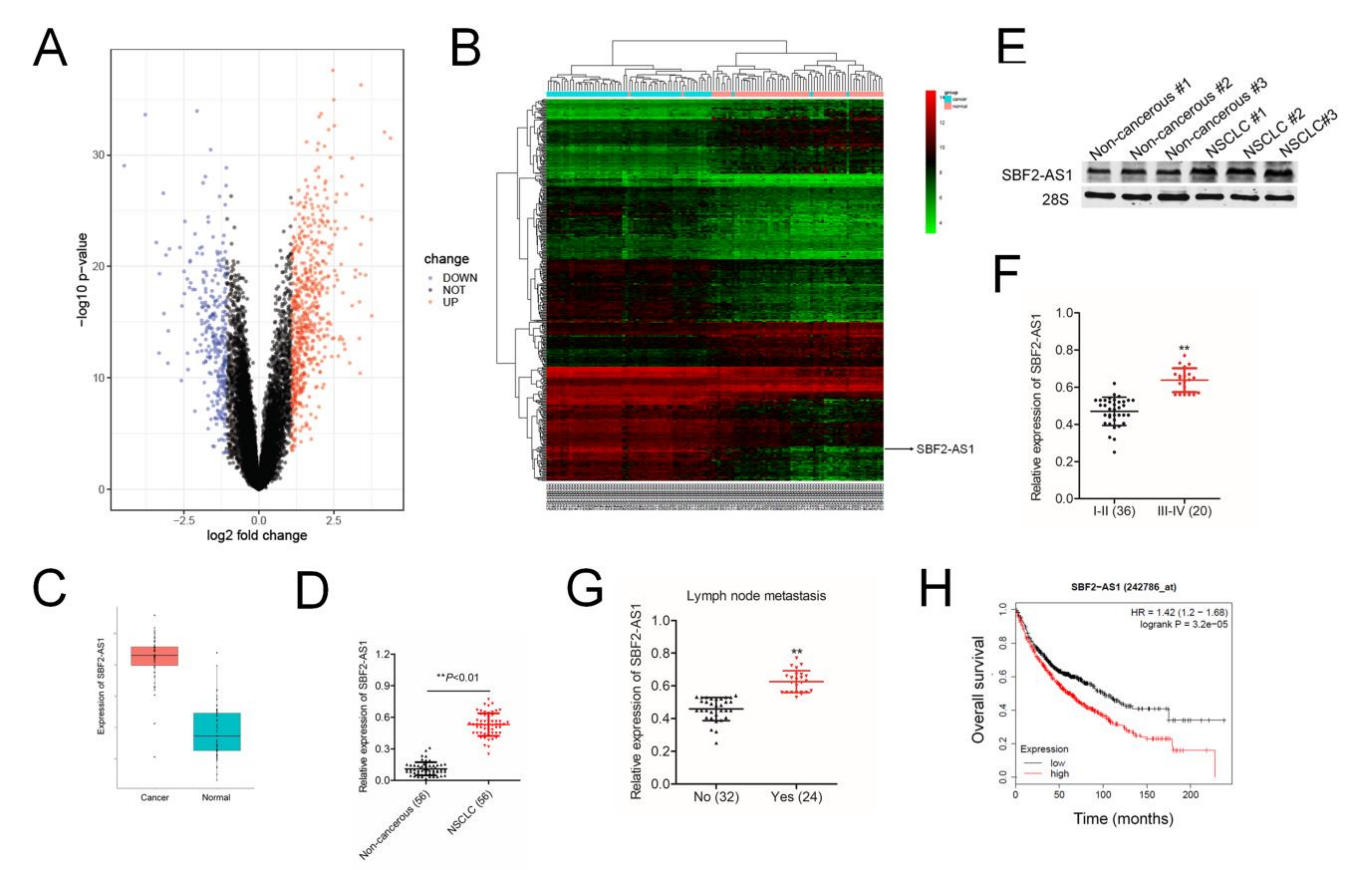
**Overexpression of SBF2-AS1 correlates with clinicopathological features and poor prognosis in patients with NSCLC.** (**A**) Volcano plot showing the upregulated and downregulated lncRNAs in the expression microarray (GSE19804 dataset). (**B**) Heatmap visualization of differentially expressed lncRNAs in normal vs lung cancer tissues (GSE19804 dataset). (**C**) Expression of SBF2-AS1 in normal and lung cancer tissues. Data represent log2 expression values. (**D**) Quantitative qRT-PCR analysis of SBF2-AS1 expression in 56 human NSCLC tissues and the paired non-cancerous tissues. ^**^P<0.01 compared with non-cancerous group. (**E**) The expressions of SBF2-AS1 in NSCLC tissues and non-cancerous tissues were detected using northern blotting assay. (**F**) The expression levels of SBF2-AS1 in patients with different stage. ^**^P<0.01 compared with I-II stage. (**G**) The expression levels of SBF2-AS1 in patients with or without metastasis. ^**^P<0.01 compared with no metastasis. (**H**) Kaplan-Meier survival analysis of overall survival rate between NSCLC patients with low or high SBF2-AS1 expression. High SBF2-AS1 was a predictor for poor overall survival of NSCLC patients as analyzed at Kaplan-Meier plotter website.

To find the potential involvement of lncRNAs in NSCLC, we performed global gene expression analysis using GEO database (the accession codes GSE19804). The upregulated lncRNAs and downregulated lncRNAs were summarized in the volcano plot (Figure 1A) and heat map (Figure 1B). Among all 5966 differential lncRNAs, there are 518 lncRNAs down-regulated and 1280 lncRNAs up-regulated in our observation on the alterations of lncRNAs expression between lung cancer and control tissues. Among these differentially expressed lncRNAs, we focus on lncRNA SBF2-AS1, due to its unknown precise biological function in NSCLC. The level of SBF2-AS1 is significantly upregulated in lung cancer from GSE19804 dataset (Figure 1C). We further detected the expression levels of SBF2-AS1 in 56 cases of clinical NSCLC samples and non-cancerous samples by using qRT-PCR. As shown in Figure 1D, SBF2-AS1 was markedly overexpressed in NSCLC tissues. Meanwhile, northern blot assay shown that SBF2-AS1 was overexpressed in human NSCLC tissue compared to in corresponding non-cancerous tissue (Figure 1E). Furthermore, the higher level of SBF2-AS1 was allied to the aggressive phenotypes of NSCLC (Figure 1F–1G and Supplementary Table 1). Finally, we confirmed that high level of SBF2-AS1 was a predictor for the poor overall survival (OS) of NSCLC patients by using Kaplan-Meier plotter (www.kmplot.com) (Figure 1H). Overall, these data showed that SBF2-AS1 was overregulated in NSCLC and might exert essential roles during the progression of NSCLC.

### SBF2-AS1 silencing restrains NSCLC cell growth and invasion ability

**Figure 2:**
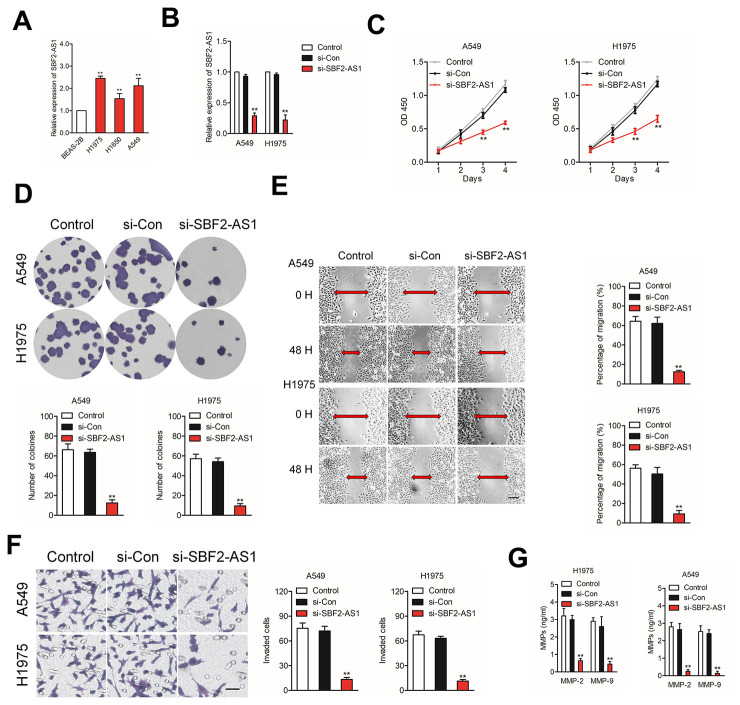
**Knockdown of SBF2-AS1 suppresses NSCLC cell proliferation, migration and invasion.** (**A**) qRT-PCR analysis of SBF2-AS1 expression in BEAS-2B and NSCLC cell lines (H1650, A549 and H1975). ^**^P<0.01 compared with BEAS-2B. (**B**) qRT-PCR analysis of SBF2-AS1 expression in A549 or H1975 cell transfected with si-Con or si-SBF2-AS1. (**C**) Cell proliferation capacity was analyzed by CCK-8 assay. (**D**) Cell proliferation capacity was analyzed by colony formation assay. (**E**) The migration capability of A549 or H1975 cell transfected with si-Con or si-SBF2-AS1 was analyzed by wound-healing assay. (**F**) The invasion capability of A549 or H1975 cell transfected with si-Con or si-SBF2-AS1 was analyzed by Transwell assay. (**G**) The expression of si-SBF2-AS1 in A549 and H1975 cell decreased secreted MMP-2/9 protein levels, as shown by ELISA. The data are presented as the mean ± SD. All *in vitro* data are representative of three independent experiments. ^**^P<0.01 compared with control.

In order to clarify the effects of SBF2-AS1, we firstly explored the level of SBF2-AS1 in non-neoplastic human bronchial epithelial cell line, BEAS-2B and NSCLC cell lines (H1975, A549 and H1650). As shown in Figure 2A, the results of qRT-PCR assay indicated that SBF2-AS1 was significantly upregulated in H1975 and A549 cell compared with that in BEAS-2B cell. Then, we knock-downed SBF2-AS1 in H1975 and A549 cell by using siRNA targeting SBF2-AS1 (Figure 2B). The CCK-8 assay indicated that NSCLC cell proliferation was significantly restrained after SBF2-AS1 silencing (Figure 2C). Consistently, the colony formation abilities of NSCLC cell were dramatically restrained after transfection with si-SBF2-AS1 (Figure 2D). Additionally, the migration and invasion abilities were markedly inhibited in A549 or H1975 cell transfected with si-SBF2-AS1 as demonstrated by wound-healing (Figure 2E) and Transwell assays (Figure 2F). Simultaneously, we observed that overexpression of lncRNA SBF2-AS1 enhanced NSCLC cell growth and invasion *in vitro* (Supplementary Figure 1). Finally, the secreted MMP-2 and MMP-9 were analyzed by ELISA. As shown in Figure 2G, the secretion of MMP-2 and MMP-9 were significantly reduced by si-SBF2-AS1 transfection in both A549 and H1975 cell. All these findings indicated the carcinogenicity of SBF2-AS1 in human NSCLC.

### SBF2-AS1 binds to miR-338-3p

**Figure 3:**
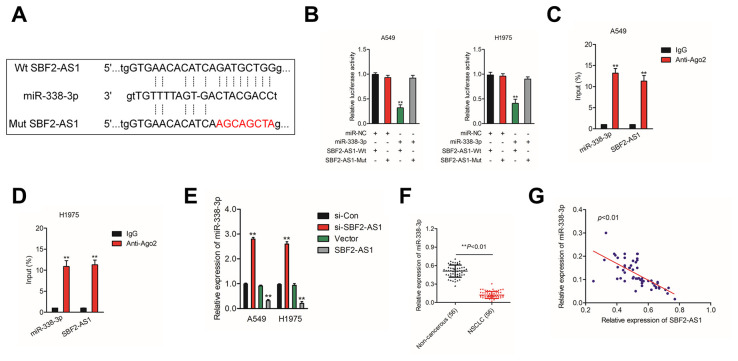
**SBF2-AS1 negatively regulates miR-338-3p expression.** (**A**) Graphical representation of the potential binding sites between SBF2-AS1 and miR-338-3p. (**B**) The relative luciferase activity in A549 or H1975 cell transfected with pmirGLO carrying SBF2-AS1-Wt or SBF2-AS1-Mut was tested. ^**^P<0.01 compared with miR-NC + SBF2-AS1-Wt. (**C**–**D**) RIP assay was performed to determine the association between SBF2-AS1 and miR-338-3p in A549 and H1975 cell. ^**^P<0.01 compared with IgG. (**E**) miR-338-3p expression level in A549 and H1975 cell transfected with SBF2-AS1 or si-SBF2-AS1 was shown. ^**^P<0.01 compared with si-Con or Vector. (**F**) miR-338-3p expression in NSCLC tissues were quantified by qRT- PCR analysis. ^**^P<0.01 compared with non-cancerous. (**G**) The negative correlation between SBF2-AS1 and miR-338-3p was displayed by Pearson’s correlation curve.

Increasing evidences have indicated that lncRNAs acts as ceRNAs competitively share miRNAs recognition sites with mRNAs to regulate the expressions of cancer-related genes. To find the potential target miRNAs of SBF2-AS1, Starbase website (http://starbase.sysu.edu.cn/) was applied to find the possible miRNAs that interact with SBF2-AS1. As show in Figure 3A, miR-338-3p has putative binding sites with SBF2-AS1. The following luciferase reporter gene analysis implied that luciferase activity in NSCLC cell transfected with pmirGLO vector carrying SBF2-AS1-Wt was remarkably reduced by miR-338-3p (Figure 3B). Moreover, the RIP assay shown that both miR-338-3p and SBF2-AS1 were enriched in Ago2 containing beads (Figure 3C–3D). In order to illuminate the relationship between miR-338-3p and SBF2-AS1, A549 and H1975 cell were transfected with si-SBF2-AS1 or pcDNA-SBF2-AS1. As shown in Figure 3E, downregulation of SBF2-AS1 increased the level of miR-338-3p and miR-338-3p was markedly suppressed in pcDNA-SBF2-AS1 transfected A549 and H1975 cell. Next, we revealed that miR-338-3p was downregulated in NSCLC tissues when comped with that in normal tissues (Figure 3F). Importantly, the level of miR-338-3p was negatively associated with SBF2-AS1 level in NSCLC tissues (Figure 3G). Altogether, these findings revealed that SBF2-AS1 bound with miR-338-3p and negatively modulated its level in NSCLC.

### Knockdown of SBF2-AS1 suppresses NSCLC cell growth and invasion via regulating miR-338-3p

**Figure 4:**
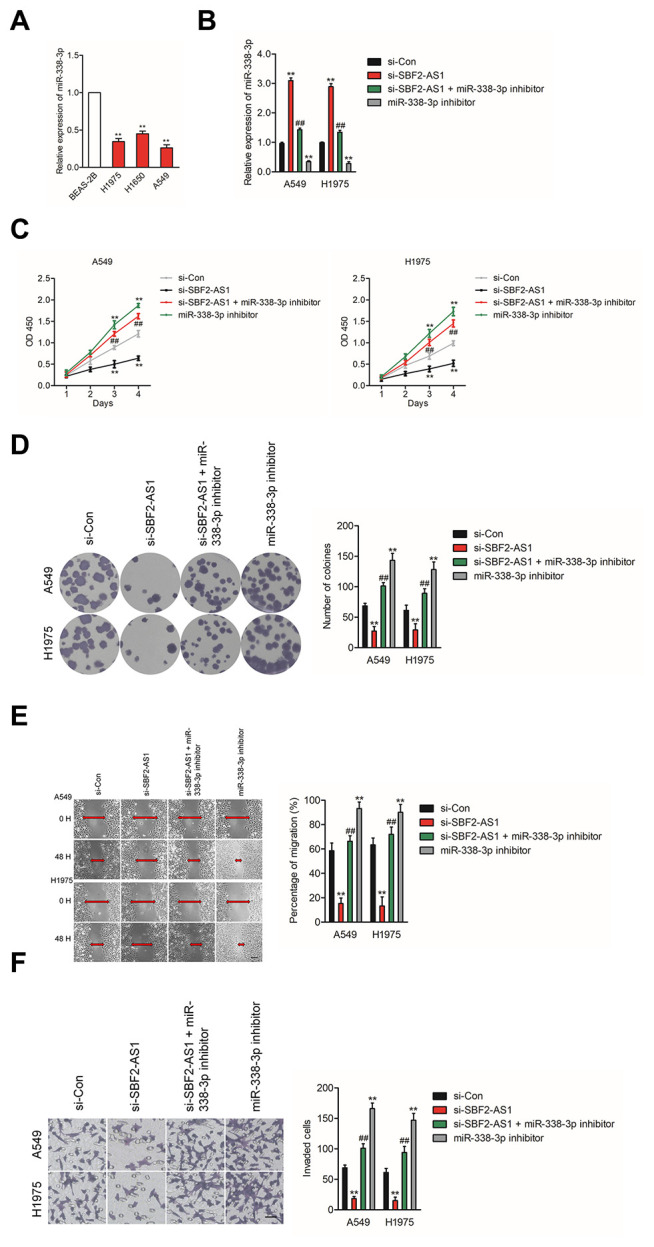
**Knockdown of SBF2-AS1 suppresses NSCLC cell proliferation and invasion by targeting miR-338-3p.** (**A**) qRT-PCR analysis of miR-338-3p expression in human bronchial epithelial cell BEAS-2B and NSCLC cell lines (A549, H1650 and H1975). ^**^P<0.01 compared with BEAS-2B. (**B**) A549 or H1975 cell was transfected with si-Con, si-SBF2-AS1 or si-SBF2-AS1 and miR-338-3p inhibitor. The expression levels of miR-338-3p in different groups were analyzed by qRT-PCR. (**C**) Cell proliferation activity of A549 or H1975 cell was assessed by CCK-8 assay. (**D**) Cell proliferation activity of A549 or H1975 cell was assessed by cell colony formation assay. (**E**) Cell migration ability of A549 or H1975 cell in different groups was analyzed by wound healing assay. (**F**) Cell invasion ability of A549 or H1975 cell in different groups was analyzed by Transwell invasion assay. The data are presented as the mean ± SD. All *in vitro* data are representative of three independent experiments. ^**^P<0.01 compared with si-Con, ^##^*P*<0.01 compared with si-SBF2-AS1.

To explore the impacts of SBF2-AS1/miR-338-3p axis on NSCLC cell, we then explored the level of miR-338-3p in normal (non-neoplastic) human bronchial epithelial cell BEAS-2B and NSCLC cell lines (A549, H1975 and H1650). The results of qRT-PCR indicated that miR-338-3p was significantly downregulated in NSCLC cell lines compared than in BEAS-2B cell (Figure 4A). Furthermore, A549 or H1975 cell were transfected with. si-SBF2-AS1 or si-SBF2-AS1 combination with miR-338-3p inhibitor (Figure 4B). The cell proliferation and colony formation assay indicated that downregulation of miR-338-3p abrogated the inhibitory impacts of si-SBF2-AS1 on the growth of NSCLC cell *in vitro* (Figures 4C–4D). Meanwhile, the wound healing and Transwell invasion assay shown that downregulation of miR-338-3p reversed abolished the suppressive impacts of si-SBF2-AS1 on the migrate and invasive abilities of NSCLC cell *in vitro* (Figures 4E–4F). All these observations suggest that downregulation of SBF2-AS1 inhibits NSCLC cell metastatic ability via regulating miR-338-3p

### SBF2-AS1 regulates the expression of ADAM17 via sponging miR-338-3p

**Figure 5:**
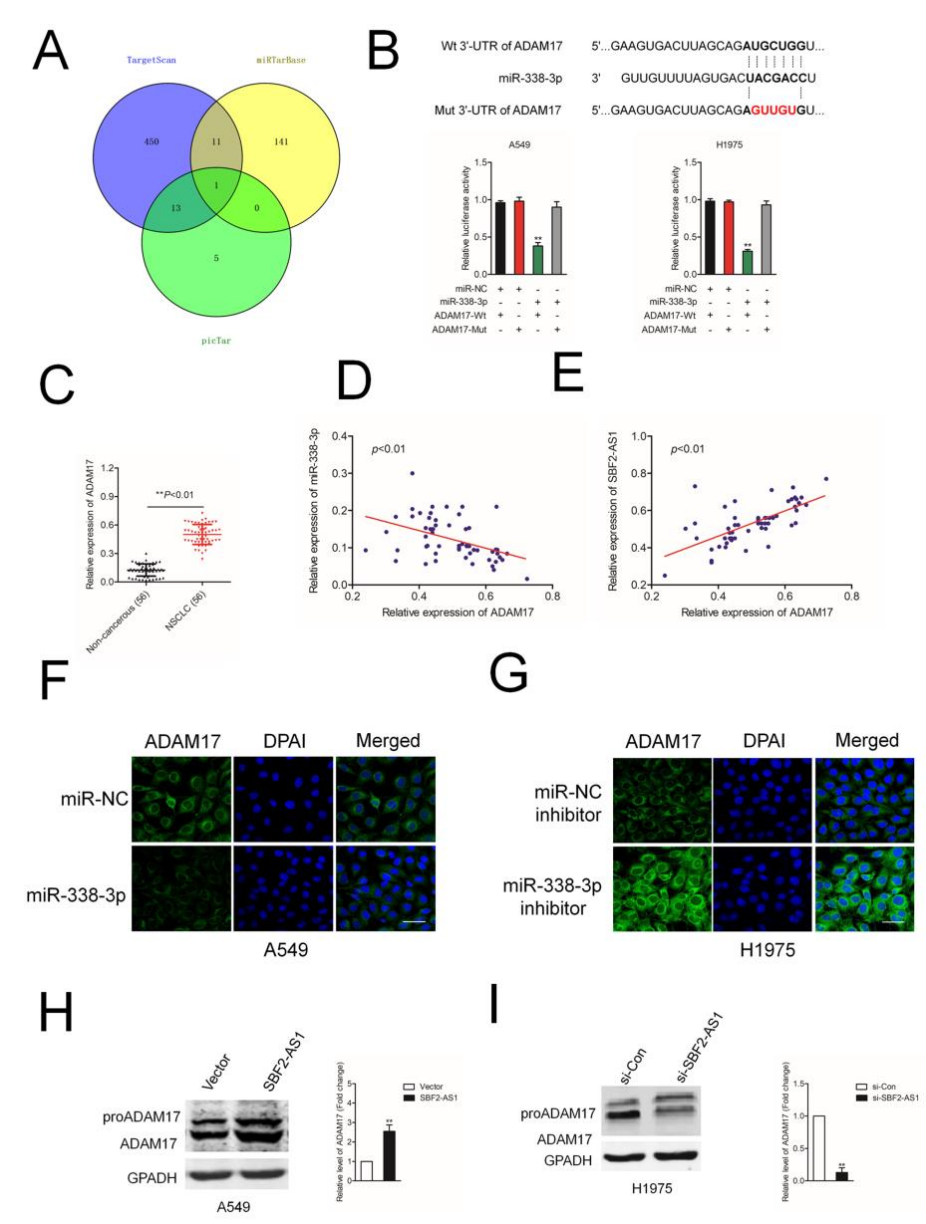
**ADAM17 is a target gene of miR-338-3p.** (**A**) Venn diagrams show the common potential target of miR-338-3p in three bioinformatics software (TargetScan, miRTarBase and picTar). (**B**) Graphical representation of the potential binding sites between miR-338-3p and ADAM17. The relative luciferase activity was tested after co-transfection with ADAM17-Wt, ADAM17-Mut and miR-338-3p. ^**^P<0.01 compared with miR-NC + ADAM17-Wt. (**C**) qRT-PCR analysis of ADAM17 expression in 56 paired human NSCLC tissues and the adjacent non-cancerous tissues. ^**^P<0.01 compared with non-cancerous. (**D**) Correlation between ADAM17 and miR-338-3p was measured by Pearson’s correlation curve. (**E**) Correlation between ADAM17 and SBF2-AS1 was measured by Pearson’s correlation curve. (**F**) The expression of ADAM17 in A549 transfected with miR-338-3p was detected using immunofluorescence staining. (**G**) The expression of ADAM17 in H1975 transfected with miR-338-3p inhibitor was detected using immunofluorescence staining. (**H**) The expression of ADAM17 in A549 transfected with si-SBF2-AS1 was detected using western blotting. (**I**) The expression of ADAM17 in H1975 transfected with SBF2-AS1 overexpression plasmid was detected using western blotting. The data are presented as the mean ± SD. All *in vitro* data are representative of three independent experiments. ^**^P<0.01 compared with si-Con or Vector.

Increasing reports demonstrate the ceRNA hypothesis, which reveals lncRNAs are involved into lncRNAs-miRNAs-mRNAs crosstalk. We firstly verified ADAM17 as a downstream target gene of miR-338-3p by using three bioinformatics software (TargetScan, miRTarBase and picTar) (Figure 5A). The following luciferase reporter gene analysis validated miR-338-3p bound with the 3’-UTR of ADAM17 (Figure 5B). Additionally, the expressions of ADAM17 in NSCLC samples were higher compared with in noncancerous tissues (Figure 5C). The Pearson correlation analysis indicated that miR-338-3p level was inversely associated with the expression of ADAM17 in NSCLC tissues (Figure 5D). Nevertheless, ADAM17 was positively related with SBF2-AS1 level in NSCLC samples (Figure 5E). In addition, we observed that the protein level of ADAM17 was significantly reduced in A549 cell transfected with miR-338-3p (Figure 5F). Nevertheless, miR-338-3p inhibitor raised the expression of ADAM17 in H1975 cell (Figure 5G). Furthermore, the protein level of ADAM17 was significantly raised in A549 cell transfected with SBF2-AS1 overexpression plasmid (Figure 5H) and si-SBF2-AS1 decreased the expression of ADAM17 in H1975 cell (Figure 5I). Early reports have indicated that ADAM8 also promotes cancer development and metastasis in a variety of tumor types [20–23]. We also analyzed the effect of SBF2-AS1 or miR-338-3p on the expression of ADAM8 in A549 cell. As shown in Supplementary Figure 2A, after transfected with si-SBF2-AS1 or miR-338-3p, the expression of ADAM8 was not significantly reduced, which indicating ADAM8 was not the potential downstream target of SBF-AS1 or miR-338-3p. In, addition, A549 cell was transfected with si-ADAM8 alone or cotransfected with si-ADAM8 and miR-338-3p. The expression of ADAM8 was assessed by western blot (Supplementary Figure 2B). After that, the migration and invasion abilities were significantly inhibited by si-ADAM8. Nevertheless, the suppressive effect of miR-338-3p on the aggressive traits was not impaired in the presence of si-ADAM8, which suggested that miR-338-3p regulated the migration and invasion of NSCLC cell in an ADAM8-independent manner (Supplementary Figure 2B).

**Figure 6:**
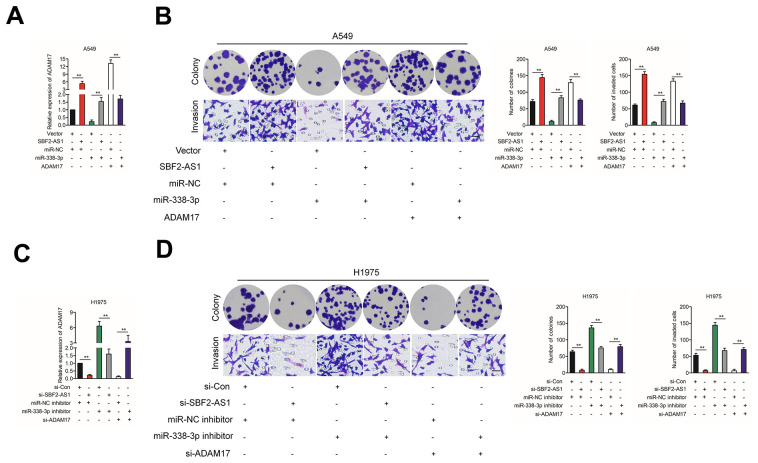
**The effect of SBF2-AS1/miR-338-3p/ADAM17 axis on NSCLC cells biological behavior *in vitro*.** (**A**) SBF2-AS1, ADAM17 and miR-338-3p were co-transfected into A549 cell, the mRNA level of ADAM17 was measured by qRT-PCR assay. (**B**) SBF2-AS1, ADAM17 and miR-338-3p were co-transfected into A549 cell, Cell growth and invasion ability were assessed by colony formation assay and Transwell assay. (**C**) si-SBF2-AS1, si-ADAM17 and miR-338-3p inhibitor were co-transfected into H1975 cell, the mRNA level of ADAM17 was measured by qRT-PCR assay. (**D**) si-SBF2-AS1, si-ADAM17 and miR-338-3p inhibitor were co-transfected into H1975 cell, cell growth and invasion ability were assessed by colony formation assay and Transwell assay. The data are presented as the mean ± SD. All *in vitro* data are representative of three independent experiments.

Finally, SBF2-AS1, ADAM17 and miR-338-3p were cotransfected into A549 cell, the protein expression of ADAM17 in A549 cell was measured by using qRT-PCR assay (Figure 6A). Then, the growth and invasion capacities were analyzed (Figure 6B). We found that overexpression of SBF2-AS1 or ADAM17 increased the colony formation and invasion abilities in A549 cell. Transfected with miR-338-3p reversed the impacts of SBF2-AS1 or ADAM17 on the aggressiveness of H1975 cell. H1975 cell was transfected with si-SBF2-AS1, si-ADAM17 and miR-338-3p inhibitor, the protein expression of in A549 cell was measured by using qRT-PCR assay (Figure 6C). Transfected with miR-338-3p inhibitor reversed the suppressive impacts of si-SBF2-AS1 or si-ADAM17 on the aggressiveness of H1975 cell (Figure 6D). Altogether, these results suggested that SBF2-AS1 regulated ADAM17 in NSCLC by serving as a molecular sponge to regulate miR-338-3p.

### ADAM17 is upregulated in NSCLC

**Figure 7:**
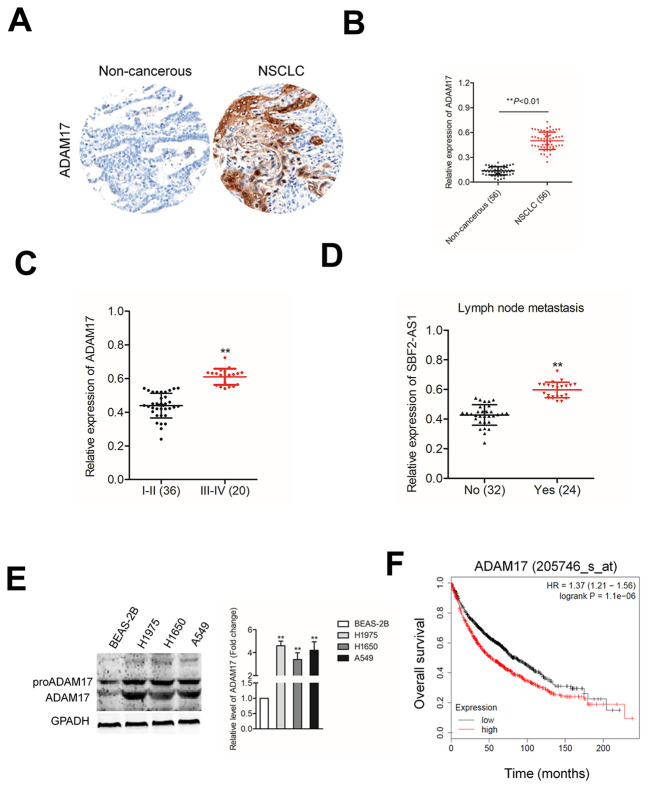
**ADAM17 is overexpressed in NSCLC.** (**A**) IHC staining of ADAM17 in NSCLC and non-cancerous tissue. (**B**) qRT-PCR analysis of ADAM17 expression in NSCLC tissues and non-cancerous tissues (**C**) The expression levels of ADAM17 in patients with different stage. ^**^P<0.01 compared with I-II stage. (**D**) The expression levels of ADAM17 in patients with or without metastasis. ^**^P<0.01 compared with no metastasis. (**E**) Western blotting analysis of ADAM17 expression in human bronchial epithelial cell BEAS-2B and NSCLC cell lines (A549, H1650 and H1975). ^**^P<0.01 compared with BEAS-2B. (**F**) High ADAM17 was a predictor for poor overall survival of NSCLC patients as analyzed using Kaplan-Meier plotter website.

To further establish the correlation between ADAM17 in NSCLC tissues, we evaluated the expressions of ADAM17 in 56 paired NSCLC tissues and corresponding normal tissues using IHC (Figure 7A). Consistently, the result of qRT-PCR assay indicated that the level of ADAM17 was higher in NSCLC tissues compared with in non-cancerous tissues (Figure 7B). Higher ADAM17 expression was associated with the metastasis and advanced tumor stage of NSCLC (Figures 7C–7D). The results of western blotting also indicated that ADAM17 was significantly overexpressed in NSCLC cell compared than in BEAS-2B cell (Figure 4E). Meanwhile, we found that high ADAM17 expression was a predictor for poor overall survival of NSCLC patients by using Kaplan-Meier plotter (www.kmplot.com) (Figure 7F). Given these results, we concluded that ADAM17 was significantly overexpressed in NSCLC.

### SBF2-AS1 silencing inhibits the growth and metastasis of NSCLC cells *in vivo*

**Figure 8:**
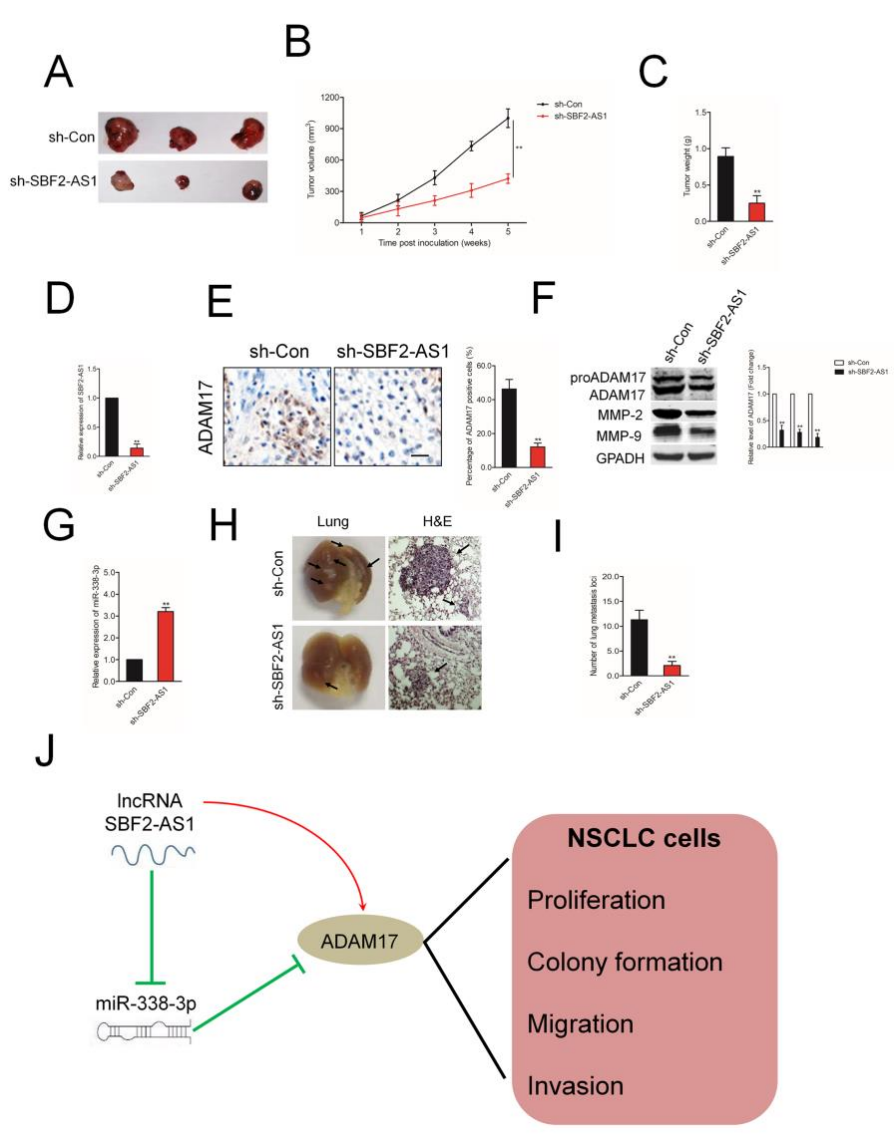
**The effect of SBF2-AS1 on tumor growth *in vivo*.** (**A**) Representative pictures of tumor xenograft. (**B**–**C**) Quantitative analysis of xenografted tumor volume and weight. (**D**) The level of SBF2-AS1 in sh-SBF2-AS1 group and sh-Con group was detected by qRT-PCR assay. (**E**) Representative IHC staining images and relative expression levels of ADAM17 positive staining in tumor sections from sh-SBF2-AS1 group and sh-Con group. (**F**) The levels of ADAM17, MMP-2 and MMP-9 in sh-SBF2-AS1 group and sh-Con group were detected by wester blotting assay. (**G**) The level of miR-338-3p in sh-SBF2-AS1 group and sh-Con group was detected by qRT-PCR assay. ^**^P<0.01 compared with sh-Con group. (**H**) Representative macroscopic pictures of mice lung, twelve weeks after inoculation (right panel). Representative photographs of H&E stained spontaneous lung metastases (left panel). (**I**) Graph displaying the total number of tumor nodules per lung in sh-Con and sh-SBF2-AS1 group. (**J**) Schematic model of SBF2-AS1-mediated the growth, migration and invasion in NSCLC cell. ^**^P<0.01 compared with sh-Con group.

In order to confirm the role of SBF2-AS1 on NSCLC cell growth *in vivo*, A549 cell was stable transfected with sh-SBF2-AS1 or sh-Con and inoculated into nude mice to construct xenograft models. As showed in Figures 8A–8C, downregulation of SBF2-AS1 drastically restrained both tumor growth and tumor weight in mice injected with sh-SBF2-AS1 transfected A549 cell. Meanwhile, when compared with the sh-Con group, the mice injected with sh-SBF2-AS1 transfected A549 cell exhibited a significantly lower relative tumor volumes (RTV) (Supplementary Figure 3). Meanwhile, the T/C %=37.6% (less than 60%). All these findings indicated that downregulation of SBF2-AS1 restrained the tumor growth of A549 cell growth *in vivo*. The levels of SBF2-AS1 in tumor tissues were further assessed using qRT-PCR assay. As shown in Figure 8D, the level of SBF2-AS1 was significantly lower in tumor tissue formed by sh-SBF2-AS1 transfected A549 cell compared with that in tumor tissue formed by sh-Con transfected A549 cell. Moreover, ADAM17 immunohistochemistry (IHC) staining assay shown that ADAM17 positive staining was suppressed in the sh-SBF2-AS1 group (Figure 8E). The results of western blotting assay indicated that the expressions of MM-P2/9 and ADAM17 were significantly reduced in sh-SBF2-AS1 group (Figure 8F). Nevertheless, the level of miR-338-3p was remarkably raised sh-SBF2-AS1 group (Figure 8G). Finally, we investigated whether downregulation of SBF-AS1 would also affect the metastatic behavior of A549 cell *in vivo*. sh-Con or sh-SBF-AS1 transfected A549 cells were inoculated into nude mice through the lateral tail vein. After twelve weeks, lung metastasis was apparent in mice injected with sh-Con transfected A549 cell (Figures 8H–8I). The numbers of lung metastasis nodules were significantly decreased in sh-SBF-AS1 group. All these observations indicated the oncogenic ability of SBF2-AS1 in NSCLC.

## DISCUSSION

Increasing researches have demonstrated that lncRNAs exert critical functions in the progression of malignant tumors, including NSCLC. For instance, lncRNA KCNQ1 Opposite Strand/Antisense Transcript 1 (KCNQ1OT1) promotes the development of NSCLC cell by modulating miR-204-5p/Autophagy Related 3 (ATG3) axis [24]. LncRNA FAM201A mediates the metastasis of lung squamous cell cancer via regulating ABCE1 expression [25]. SBF2-AS1, a recently identified lncRNA, has been proved to be overexpressed and exerts a critical role in several cancers. Upregulated lncRNA SBF2-AS1 enhances the proliferation of esophageal squamous cell carcinoma [14]. In colorectal cancer, lncRNA SBF2-AS1 promotes colon cancer cell growth and invasion via suppressing miR-619-5p activity and facilitating Histone Deacetylase 3 (HDAC3) expression [8]. Early investigation has indicated that higher level of SBF2-AS1 is related to poor clinical outcomes in patients with NSCLC [26]. Consistent with the previous reports, in our current study, we proved that SBF2-AS1 was remarkably overexpressed in NSCLC samples as well as cell lines. In addition, high level of SBF2-AS1 was positively connected to distant metastasis and advanced stage as well as poor clinical outcome of patients with NSCLC. Furthermore, downregulation of SBF2-AS1 impaired the growth, migration and invasion of NSCLC cell *in vitro*, and repressed NSCLC cell growth *in vivo*. All data illustrated the tumorigenicity of SBF2-AS1 in NSCLC.

Previous investigations proved that lncRNAs act as miRNAs sponges to modulate miRNAs. For example, UICLM, LINC00657 and SNHG15 were reported to facilitate cancer development via serving as miRNAs sponges in colorectal carcinoma, esophageal squamous cell carcinoma and papillary thyroid carcinoma [3, 27, 28]. In our study, the bioinformatics prediction tools revealed that miR-338-3p had potential binding sites between SBF2-AS1. Simultaneously, the following luciferase reporter assay and RIP assay suggested a directly interaction between miR-338-3p and SBF2-AS1. Moreover, we found that SBF2-AS1 level was negatively related with the expression of miR-338-3p in NSCLC samples. It has been reported that down-regulation of miR-338-3p indicates a poor prognosis of patients with epithelial ovarian cancer (EOC) [29]. In addition, miR-338-3p modulates the growth, apoptotic and migration ability of colon cancer cell through regulating MACC1 [30]. In order to study the biological function of SBF2-AS1 and miRNA-338-3p in NSCLC cell, several rescues experiments were carried out. We revealed that although knockdown of SBF2-AS1 repressed the growth and aggressive traits of NSCLC cell, miRNA-338-3p inhibitor reversed the effects of SBF2-AS1-specific siRNA. These results implied that SBF2-AS1 acted as a sponge of miR-338-3p in human NSCLC.

MiRNAs are a class of non-coding RNAs that function as post-transcriptional regulators through interacting with the 3’-UTR of mRNAs. Our study demonstrated that miR-98-5p bound to the 3’-UTR of ADAM17 and reduced the expression of ADAM17 in NSCLC cell. The Pearson analysis indicated there was an inversely relationship between miR-338-3p and ADAM17 in NSCLC tissues. However, ADAM17 level was positively related with the level of SBF2-AS1 in NSCLC tissues. The expression and activity of ADAM17 increase under some pathological conditions such as breast cancer and lung cancer [31–33]. Consistent with previous findings, our results uncovered that ADAM17 was markedly overregulated in human NSCLC tissues compared with in non-cancerous tissues. The level of ADAM17 was also higher in NSCLC cell lines compared with that in non-neoplastic human bronchial epithelial cell BEAS-2B. Additional, our study indicated that SBF2-AS1 positively regulated the level of ADAM17 through sponging for miR-338-3p in NSCLC cell. Importantly, rescues experiments indicated that dysregulations of SBF2-AS1, miR-338-3p and ADAM17 were allied to the progression of NSCLC cell.

Our study demonstrated that lncRNA SBF2-AS1 acted as a ceRNA to modulate the expression of ADAM17 through sponging miR-338-3p and exerted oncogenic roles during the progression of NSCLC (Figure 8J). This research revealed the critical roles of lncRNA SBF2-AS1/miR-338-3p/ADAM17 axis in the growth and aggressive phenotypes of NSCLC, which might be served as a therapeutic target for NSCLC.

## MATERIALS AND METHODS

### Bioinformatics analysis

GSE dataset (GSE19804) containing lncRNAs expression profiles were retrieved from the Gene Expression Omnibus (GEO) database (http://www.ncbi.nlm.nih.gov/geo/). The R software package was used to detect the differential expression of lncRNAs and to construct the figures.

### NSCLC samples and cell lines

56 cases of NSCLC samples and corresponding non-cancerous samples were collected from NSCLC patients in the Affiliated Hospital of Southwest Medical University. Patients received no treatments before this study. Written informed consent was obtained from participants. All tissues were conserved in liquid nitrogen. This study was approved by the Ethics Committee of the Affiliated Hospital of Southwest Medical University. The clinicopathological features of patients were shown in Supplementary Table 1.

### Cell culture

NSCLC cell lines (A549, H1650 and H1975), non-neoplastic human bronchial epithelial cell line BEAS-2B were purchased from the Chinese Academy of Sciences (Shanghai, China). Cells were cultured in RPMI-1640 (Thermo Fisher Scientific, Waltham, MA, USA) supplemented with 10% FBS and 100 U/ml penicillin/streptomycin and maintained in an incubator with 5% CO_2_ and 95% air at 37°C.

### Cell transfections

The pcDNA3.1 vector carrying SBF2-AS1 (SBF2-AS1), pcDNA3.1 vector carrying ADAM17 (ADAM17) or empty vector (vector) were obtained from RiboBio (Guangzhou, Guangdong, China). MiR-338-3p mimics, miRNA negative control (miR-NC), miR-338-3p inhibitor, miR-NC inhibitor, small interfering RNA (siRNA) targeting SBF2-AS1 or ADAM17 (si-SBF2-AS1, si-ADAM17) and scramble control siRNA (si-Con) were purchased from RiboBio. 2×10^4^ A549 or H1975 cells were transfected with 100 μmol/L miRNAs, or 5 μg pcDNA3.1, or 50 nM siRNA using 7.5 μl of Lipofectamine 3000 (Thermo Fisher Scientific) in 125 μl of Opti-MEMTM medium combination with 5 μl of p3000 for twenty-four hours. For functional assay *in vivo*, sh-SBF2-AS1 and sh-Con were purchased from RiboBio and constructed into A549 cell. The cDNA sequences of SBF2-AS1 were constructed by Shanghai Jima Pharmaceutical Technology Co., Ltd., (Shanghai, China) and were subcloned into pLKO.1 lentivirus vector (Addgene). pLKO.1 empty vector was used as negative control plasmid. Then, lentivirus plasmid was transfected into HEK-293T cell along with lentivirus packaging plasmids (psPAX2 and pMD2.G, Addgene). 72 hours after transfection, cell supernatants were collected and A549 cell was infected with lentivirus, followed by the screening of 1.5 μg/ml puromycin (Sigma). After 7 days, stable lentivirus transfected A549 cell lines were obtained.

### Luciferase reporter gene assay

The fragment of SBF2-AS1 possessing the miR-338-3p binding sites was inserted into pmirGLO luciferase vector (Promega, Madison, WI, USA) to construct SBF2-AS1-wild-type (SBF2-AS1-Wt). The corresponding mutant of miR-338-3p binding sites was constructed to form SBF2-AS1-mutated-type (SBF2-AS1-Mut). The 3’-UTR fragment of ADAM17 or its mutant of the miR-338-3p binding sites was inserted into pmirGLO to form ADAM17-3’-UTR-Wt or ADAM17-3’-UTR-Mut, respectively. The pmirGLO vector and miR-338-3p were transfected into A549 or H1975 cell using Lipofectamine 3000. After 48 hours, the luciferase activities were measured using Luciferase Reporter Assay System (Promega).

### RNA immunoprecipitation (RIP) assay

Cells were lysed using RIP lysis buffer (Thermo Fisher Scientific) and the lysates were conjugated with anti-Argonaute2 (Ago2) antibody (Millipore, Braunschweig, Germany) or negative control anti-IgG in magnetic bead. Finally, the retrieved RNA was detected using qRT-PCR analysis.

### Counting kit-8 (CCK-8) assay

Cells (1 × 10^4^) were plated into 96-well plates for 24, 48, 72 or 96 hours. Cell viability was measured using CCK-8 kit (Beyotime Biotechnology, Nanjing, Jiangsu, China).

### Colony formation assay

Cells (1×10^3^) were cultured into 6 well plates. After culturing for two weeks, the cell colonies in plates were fixed using methanol and stained with 1% crystal violet solution for 15 min [16, 17]. The number of cell colonies was counted under a microscope.

### Wound healing assay

A549 or H1975 cells (1 × 10^5^) were seeded into 6-well plates. Cells were treated with mitomycin (10 μg/ml) for 1 h at 37°C and then washed twice with PBS [18]. Then, an artificial wound was made by utilizing a 200 μl sterile tip. A549 or H1975 cells were maintained for 48 hours and the wounded areas were photographed at 0 hour and 48 hours under inverted microscope. Percentage of migration = (0 h width of scratch - 48 h width of scratch)/0 h width of scratch × 100%.

### Transwell invasion

Cell invasion was detected using Transwell chambers coated with Matrigel (BD). In the upper chamber, cells (5×10^4^) were maintained with serum-free media. 600 μl DMEM containing 20% FBS was added into the lower chamber of Transwell. After 18 hours, the number of invaded cell was counted under inverted microscope.

### Immunoblotting

Total proteins were extracted using RIPA buffer (Thermo Fisher Scientific). 25 μg of proteins were loaded onto 8% SDS-PAGE and transferred to the PVDF membranes (Millipore, Braunschweig, Germany). After blocking with 5% BSA, the PVDF membrane was incubated with GAPDH (1:1000, Abcam, Cambridge, UK) or ADAM17 antibody (1:1000, Abcam, Cambridge, UK) overnight. After washed with PBST, the PVDF membrane was incubated with secondary antibody (1:10000, Beyotime Biotechnology, Nanjing, Jiangsu, China) for 2 hours. Finally, the bands were detected using ECL kit (GE Healthcare, USA).

### Northern blotting

20 μg RNA was electrophoresed on a 10% NovexTM TBE-Urea Gel (Thermo Fisher Scientific) and transferred to a Hybond-N+ membrane using a semi-dry electroblotter at 400 mA for 30 min, followed by UV crosslinking. Oligo DNA probes were labeled with gamma [32P]-ATP by using a MEGALABEL Kit (Thermo Fisher Scientific). Hybridization was conducted in hybridization buffer at 65 °C overnight. The membrane was washed in 2x SSC and 0.5% SDS for 30 min and in 0.2x SSC and 0.5% SDS at 65 °C for 30 min. Finally, the signals were detected with a BAS-3000 image-analyzer (GE Healthcare).

### qRT-PCR

Total RNAs were extracted utilizing Trizol kit (Thermo Fisher Scientific). RNA (1 μg) was reversely transcribed into cDNA using the PrimeScript RT reagent kit (TakaraBio, Tokyo, Japan) and a TaqMan miRNA reverse transcription kit (Applied Biosystems, Foster City, CA, USA). qRT-PCR was carried out using SYBR Premix Ex Taq™ kit (TakaraBio) and miRNA-specific TaqMan miRNA assay kit (Applied Biosystems) on the Applied Biosystems 7500 Sequence Detection system (Applied Biosystems). The primers were as follows: SBF2-AS1 (forward primer: 5’-AGTTGAGGGTCAAGCTGCTC-3’; reserve primer: 5’-TAGAGAGCCAGGGGATG-3’), ADAM17 (forward primer: 5’-GTGGATGGTAAAAACGAAAGCG-3’; reserve primer: 5’-GGCTAGAACCCTAGAGTCAGG-3’), U6 (forward primer: 5’-CGCTTCGGCAGCACATATAC-3’; reverse primer: 5’-TTCACGAATTTGCGTGTCAT-3’), GAPDH (forward primer: 5’-AGGTCGGTGTGAACGGATTTG-3’; reverse primer: 5’-TGTAGACCATGTAGTTGAGGTCA-3’). GAPDH was used as internal control for genes and U6 was used as internal control for miR-338-3p. The comparative cycle threshold (Ct) method was used to calculate the level of miRNA or genes by calculating the 2(^-ΔΔCt^).

### Immunofluorescence

A549 or H1975 cells were grown on coverslips in 24-well plates. After washing in PBS, cells were fixed with 4% paraformaldehyde and permeabilized with 0.2% Triton X-100/PBS. Cells were incubated with ADAM17 antibody (1:500, Abcam, Cambridge, UK) followed by incubation with FITC-conjugated anti-rabbit secondary antibody. Cell nuclei were visualized with DAPI (Sigma). The fluorescence signal was examined under a fluorescence microscope (Olympus, Tokyo, Japan).

### Enzyme-linked immunosorbent assay (ELISA)

A549 or H1975 cells (5 × 10^4^ per well) in 96-well plates were transfected with si-Con or si-SBF2-AS1. Culture medium was collected, and secreted matrix metalloproteinase-2 (MMP-2) and MMP-9 were detected with the human MMPs ELISA kit (R&D Systems, Minneapolis, MN, USA).

### Kaplan-Meier (KM) Plotter analysis

To analyze the overall survival of NSCLC patients with low expression or high expression of SBF2-AS1, KM Plotter was used online (http://kmplot.com) based on the lung cancer database by selecting the non-commercial spotted platform, and patients were split by auto select best cutoff.

### *in vivo* assay

BALB/C nude mice were bought from Shanghai SLAC Laboratory Animal Center (Shanghai, China). SBF2-AS1 knockdown lentivirus (sh-SBF2-AS1) or empty lentivirus control (sh-Con) transfected A549 cells (1×10^7^) were suspended in 100 μl of FBS-free RPMI-1640 and subcutaneously implanted into nude mice. Tumor volume (TV) was calculated each week. TV = 0.5×length×width^2^. Relative tumor volume (RTV)=Vt/V0. Vt is the tumor volume on each measurement and V0 is the tumor volume at the beginning. Treatment/Control (T/C) %= RTV in sh-SBF2-AS1 group/RTV in sh-Con group×100. T/C%<60% indicates the significant inhibitory role of sh-SBF2-AS1 in the growth of A549 cell *in vivo*. Immunohistochemical (IHC) staining of ADAM17 was conducted using the xenograft tumor tissues. For IHC scoring, weighted score was computed that represented the positive staining of ADAM17. Percent positive staining was categorized as follows: 0, <5%; 1, 5-25%; 2, 26-50%; 3, 51-75%, and 4, >75% [19]. For the experimental metastasis mouse xenograft model, sh-Con or sh-SBF2-AS1 transfected A549 cells were inoculated into BALB/C nude mice via the tail vein. After twelve weeks, the mice were sacrificed, and the lungs were fixed in formalin and subjected for with hematoxylin-eosin (H&E) staining. All animal procedures were approved by the Affiliated Hospital of Southwest Medical University.

### Statistical analysis

All data were shown as mean ± standard deviation (SD) and calculated using GraphPad Prism 8.0. All data are from three independent experiments. The differences were calculated using Student’s t-test or one-way ANOVA followed by Tukey’s post-hoc test. Spearman correlations among variables were calculated. Survival curves were plotted using Kaplan-Meier method and calculated using the log-rank test. The Mann-Whitney U test was used to determine the association between SBF2-AS1 expression and clinicopathological parameters in patients with NSCLC. *P*<0.05 is considered statistical significance.

## Supplementary Material

Supplementary Figures

Supplementary Table 1
