# Supplementary material for: Long noncoding RNA SBF2-AS1 contributes to the growth and metastatic phenotypes of NSCLC via regulating miR-338-3p/ADAM17 axis
Source: Aging (Albany NY). 2020 Sep 25;12(18):17902–20. doi: 10.18632/aging.103332 (PMC7585082; doi:10.18632/aging.103332)
Supplement: Supplementary Figures [file aging-12-103332-s002..pdf]

SUPPLEMENTARY FIGURES

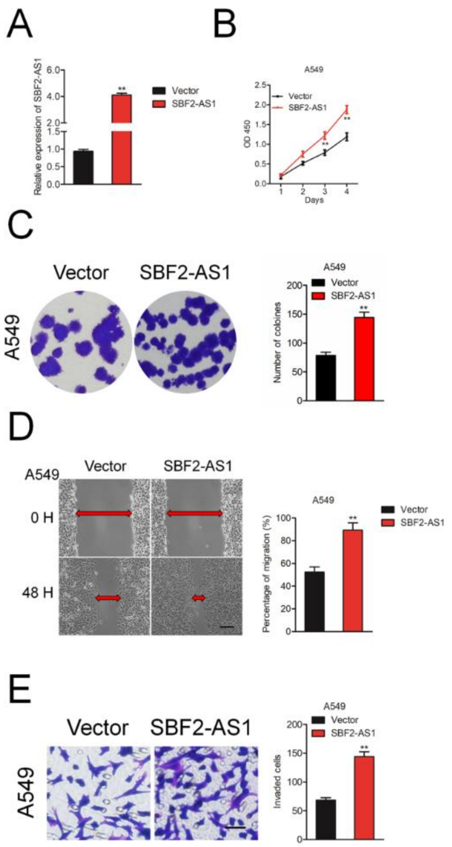

Supplementary Figure 1. SBF2-AS1 promotes NSCLC cell proliferation and invasion. (**A**) A549 cells were transfected with vector or SBF2-AS1. The expression levels of SBF2-AS1 in different groups were analyzed by qRT-PCR. (**B**) Cell proliferation activity of A549 cells was assessed by CCK-8 assay. (**C**) Cell proliferation activity of A549 cells was assessed by cell colony formation assay. (**D**) Cell migration ability of A549 cells in different groups was analyzed by wound healing assay. (**E**) Cell invasion ability of A549 cells in different groups was analyzed by Transwell invasion assay. $^{**}P$<0.01 compared with vector.

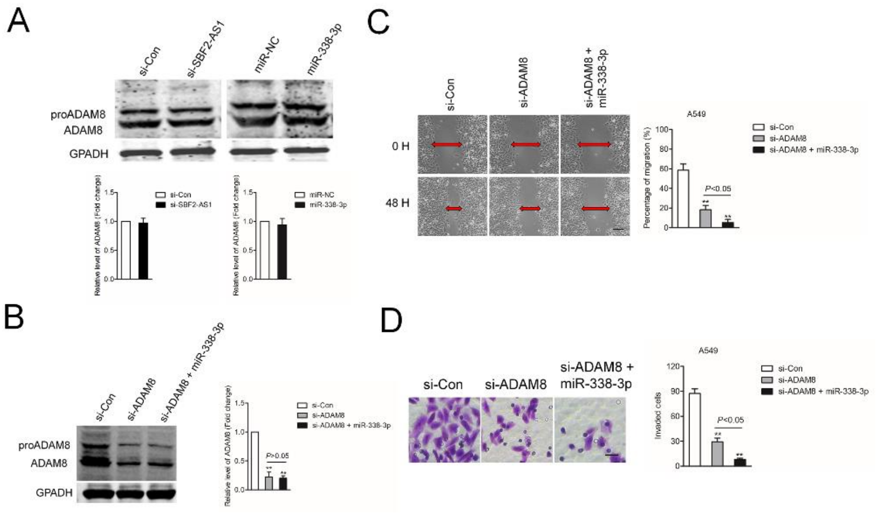

Supplementary Figure 2. miR-338-3p inhibits the migration and invasion of si-ADAM8 transfected A549 cell. (**A**) A549 cells were transfected with si-SBF2-AS1 or miR-338-3p. The expression levels of ADAM8 in different groups were analyzed by western blotting. (**B**) A549 cells were transfected with si-ADAM alone or cotransfected with si-ADAM8 plus miR-228-3p. The expression levels of ADAM8 in different groups were analyzed by western blotting. (**C**) Cell migration of A549 cells was assessed by wound healing assay. (**D**) Cell invasion ability of A549 cells in different groups was analyzed by Transwell invasion assay. ***P*<0.01 compared with si-Con.

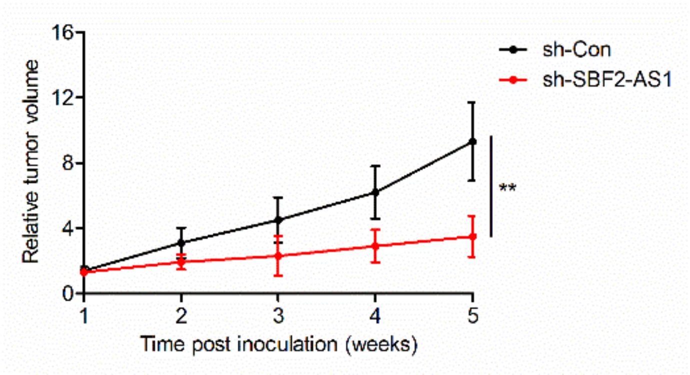

Supplementary Figure 3. Relative tumor volumes (RTVs) were measured in the different groups. ***P*<0.01 compared with sh-Con.
