# Supplementary material for: Long noncoding RNA SBF2-AS1 contributes to the growth and metastatic phenotypes of NSCLC via regulating miR-338-3p/ADAM17 axis
Source: Aging (Albany NY). 2020 Sep 25;12(18):17902–20. doi: 10.18632/aging.103332 (PMC7585082; doi:10.18632/aging.103332)
Supplement: Supplementary Table 1 [file aging-12-103332-s001..pdf]

SUPPLEMENTARY TABLE

Supplementary Table 1. Characteristics of patients with NSCLC in the present study.

Variables	SBF2-AS1		*P*-value	miR-338-3p		*P*-value	*ADAM17*		*P*-value
	High (n)	Low (n)		High (n)	Low (n)		High (n)	Low (n)	
**Age (years)**			0.21			0.44			0.18
<50	18	20		14	24		16	22	
>50	14	4		12	6		12	6	
**Gender**			0.36			0.51			0.28
Male	20	15		16	19		15	20	
Female	12	9		10	11		13	8	
**Tumor size**			0.078			0.113			0.084
≤3 cm	21	16		17	20		15	22	
>3 cm	11	8		9	10		13	6	
**Differentiation**			0.104			0.13			0.141
High	10	4		6	8		0	14	
Medium	13	8		12	9		13	8	
Low	9	12		8	13		15	6	
**LNM**			<0.01			<0.01			<0.01
Yes	22	2		6	18		16	8	
No	10	22		20	12		12	20	
**TNM stage**			<0.01			<0.01			<0.01
I-II	20	16		22	14		12	24	
II-IV	12	8		4	16		16	4	
